# Acute arterial baroreflex‐mediated changes in plasma catecholamine concentrations in a chronic rat model of myocardial infarction

**DOI:** 10.14814/phy2.12880

**Published:** 2016-08-05

**Authors:** Toru Kawada, Tsuyoshi Akiyama, Meihua Li, Can Zheng, Michael J. Turner, Mikiyasu Shirai, Masaru Sugimachi

**Affiliations:** ^1^ Department of Cardiovascular DynamicsNational Cerebral and Cardiovascular CenterOsakaJapan; ^2^ Department of Cardiac PhysiologyNational Cerebral and Cardiovascular CenterOsakaJapan

**Keywords:** Arterial pressure, carotid sinus baroreflex, myocardial infarction, norepinephrine, open‐loop analysis

## Abstract

While it may be predictable that plasma norepinephrine (NE) concentration changes with efferent sympathetic nerve activity (SNA) in response to baroreceptor pressure inputs, an exact relationship between SNA and plasma NE concentration remains to be quantified in heart failure. We examined acute baroreflex‐mediated changes in plasma NE and epinephrine (Epi) concentrations in normal control (NC) rats and rats with myocardial infarction (MI) (*n* = 6 each). Plasma NE concentration correlated linearly with SNA in the NC group (slope: 2.17 ± 0.26 pg mL^−1^ %^−1^, intercept: 20.0 ± 18.2 pg mL^−1^) and also in the MI group (slope: 19.20 ± 6.45 pg mL^−1^ %^−1^, intercept: −239.6 ± 200.0 pg mL^−1^). The slope was approximately nine times higher in the MI than in the NC group (*P* < 0.01). Plasma Epi concentration positively correlated with SNA in the NC group (slope: 1.65 ± 0.79 pg mL^−1^ %^−1^, intercept: 115.0 ± 69.5 pg mL^−1^) and also in the MI group (slope: 7.74 ± 2.20 pg mL^−1^ %^−1^, intercept: 24.7 ± 120.1 pg mL^−1^). The slope was approximately 4.5 times higher in the MI than in the NC group (*P *<* *0.05). Intravenous administration of desipramine (1 mg kg^−1^) significantly increased plasma NE concentration but decreased plasma Epi concentration in both groups, suggesting that neuronal NE uptake had contributed to the reduction in plasma NE concentration. These results indicate that high levels of plasma catecholamine in MI rats were still under the influence of baroreflex‐mediated changes in SNA, and may provide additional rationale for applying baroreflex activation therapy in patients with chronic heart failure.

## Introduction

Norepinephrine (NE) is the most important neurotransmitter at sympathetic nerve terminals. Its action is terminated by the removal of NE from the synaptic cleft via neuronal and extraneuronal uptake mechanisms (Nicholls 1994; Eisenhofer et al. 1996; Shimizu et al. 2010). A fraction of synaptic NE is diffused into the bloodstream and can be measured as plasma NE (Goldstein et al. 1983). While it may be predictable that plasma NE concentration reflects efferent sympathetic nerve activity (SNA), an exact relationship of plasma NE concentration versus SNA during acute baroreflex‐mediated changes remains to be elucidated. Since plasma NE concentration is not always measured simultaneously with SNA, knowledge about the types of relationship between the two quantities (e.g., linear or logarithmic) would help translating plasma NE concentration into SNA, and vice versa. Previous studies indicate that arterial pressure (AP) increases with logarithm of exogenously infused dose of NE (Kawada et al. 2014a, 2015) or logarithm of plasma NE concentration during electrical stimulation of the spinal cord (Yamaguchi and Kopin 1980). In contrast, AP changes nearly linearly with SNA during acute baroreflex‐mediated changes (Kawada et al. 2010; Kawada and Sugimachi 2016). If these results are put together, plasma NE concentration expressed in a logarithmic scale, rather than a normal scale, should linearly correlate with SNA. Contrary to this prediction, our previous study revealed an approximately positive linear relationship of endogenous plasma NE concentration expressed in a normal scale versus SNA during acute baroreflex‐mediated changes (Kawada et al. 2014a).

It remains unanswered whether the positive linear relationship between SNA and plasma NE concentration is also applicable to a diseased condition of excess sympathoexcitation, as observed in chronic heart failure. Since plasma NE concentration can increase several times higher in patients with heart failure than without (Viquerat et al. 1985), it is possible that plasma NE concentration can no longer change linearly with SNA due to a saturation phenomenon. Another factor that needs to be considered is neuronal NE uptake. An impairment of neuronal NE uptake can increase the diffusion of NE from the synaptic cleft into the bloodstream and change the relationship of plasma NE concentration versus SNA (Kawada et al. 2014a). Decreased efficiency of neuronal NE uptake contributes to increased cardiac adrenergic drive in patients with congestive heart failure (Eisenhofer et al. 1996). Furthermore, a neuronal NE uptake transporter can reverse its action under myocardial ischemia, leading to nonexocytotic NE release that is independent of SNA (Schömig et al. 1987; Kawada et al. 2000; Akiyama and Yamazaki 2001). While changes in neuronal NE uptake may primarily occur in the cardiac sympathetic nerve, dysfunction of neuronal NE uptake has been also reported in small arteries obtained by gluteal biopsies in patients with chronic heart failure (Hillier et al. 1999), suggesting a possible impairment of neuronal NE uptake in systemic vasculature. Accordingly, the first purpose of this study was to investigate the relationship between SNA and plasma NE concentration during acute baroreflex‐mediated changes in a chronic rat model of myocardial infarction (MI). For comparison, changes in plasma epinephrine (Epi) concentration were also analyzed. The second purpose was to examine the effect of neuronal NE uptake blockade on plasma NE concentration in MI rats. We hypothesized that, if neuronal NE uptake is already impaired in systemic vasculature of MI rats, then blocking the neuronal NE uptake would little affect plasma NE concentration.

## Materials and Methods

Animal care was provided in strict accordance with the *Guiding Principles for the Care and Use of Animals in the Field of Physiological Sciences*, approved by the Physiological Society of Japan. All protocols were reviewed and approved by the Animal Subject Committee of the National Cerebral and Cardiovascular Center.

### Surgical preparation

Male Sprague–Dawley rats were divided into normal control (NC) and MI groups. In the MI group, the left coronary artery was ligated under halothane anesthesia at the age of 8 weeks according to a previously established procedure in our laboratory (Li et al. 2004, 2014; Kawada et al. 2010, 2014b, 2015). Butorphanol tartrate was given intramuscularly at the end of the surgery to provide analgesia. In order to make the situation of the MI rat comparable to our previous study (Li et al. 2004), we implanted a dummy transmitter intraperitoneally 1 week following the coronary artery ligation under halothane anesthesia (Kawada et al. 2014b). About 8–9 weeks after MI, the surviving rats underwent an acute baroreflex experiment as described below (*n* = 6), and the results were compared with the NC rats without sham surgery (*n* = 6). One rat that underwent the left coronary ligation, did not develop significant biventricular remodeling, and was included in neither the MI nor the NC group. Information on this rat is provided in the limitation section for discussion on possible long‐term effects of past surgery.

The acute baroreflex experiment was conducted similarly to our previous study (Kawada et al. 2014a). Briefly, the rat was anesthetized with an intraperitoneal injection (2 mL kg^−1^) of a mixture of urethane (250 mg mL^−1^) and *α*‐chloralose (40 mg mL^−1^). Artificial ventilation with oxygen‐supplied room air was performed through a tracheal tube. A maintenance dose of the anesthetics (the anesthetic mixture diluted to one‐eighteenth concentration with saline, 2 mL kg^−1^ h^−1^) was administered through a catheter inserted into the right femoral vein. Another venous catheter was inserted into the left femoral vein and advanced into the inferior vena cava to measure central venous pressure. An arterial catheter was inserted into the right femoral artery to measure AP and heart rate. Another arterial catheter was inserted into the left common carotid artery and advanced into the aorta to obtain arterial blood samples. Through a left flank incision, SNA was recorded from a postganglionic branch of the splanchnic sympathetic nerve. A biosignal amplifier (AB‐610J, Nohon Kohden, Japan) amplified the raw signal by 200,000 times (1 V/5 *μ*V) with a bandpass filter between 150 and 1000 Hz. The signal was then full‐wave rectified and low‐pass filtered with a cut off frequency of 30 Hz by use of analog circuits. The aortic depressor nerves and the vagal nerves were sectioned to minimize confounding reflex effects from aortic baroreceptors and cardiopulmonary receptors. Bilateral carotid sinus regions were isolated (Shoukas et al. 1991; Sato et al. 1999) to control carotid sinus pressure (CSP). Heparin sodium (100 U kg^−1^) was given intravenously to prevent blood coagulation. After completing the above surgery, a 60‐min stabilization period was allowed before data acquisition.

### Protocol

Carotid sinus pressure was first decreased to 60 mmHg for 4 min, and then increased to 100, 120, 140, and 180 mmHg in a staircase manner. The step duration was 120 sec, and at the 100th sec of each step, arterial blood (0.2 mL) was collected in a sample tube in exchange of an equivalent volume of heparinized saline (Kawada et al. 2014a). To avoid contamination of the blood within the catheter, an initial 0.2 mL blood was withdrawn into a temporary syringe prefilled with 0.2 mL heparinized saline, the following 0.2 mL blood was taken into a collecting syringe, and then the initial blood, admixed with heparinized saline, was returned into the artery. A new collecting syringe was used for each sampling. The blood samples were immediately iced at 4°C for later plasma catecholamine measurements. After collecting data under the control condition, a neuronal NE uptake blocker desipramine (1 mg kg^−1^, bolus) was administered intravenously. Twelve minutes later, the staircase‐wise CSP input was repeated and the blood samples were collected (Kawada et al. 2014a). At the end of the experiment, a ganglionic blocker hexamethonium bromide was intravenously administered (60 mg kg^−1^, bolus) to silence SNA, which enabled confirmation that the nerve fibers we were recording were mostly postganglionic.

### Data analysis

Blood samples were centrifuged and measured for plasma NE and Epi concentrations using a high‐performance liquid chromatography system (Eicom, Kyoto, Japan) via an alumina adsorption procedure (Kawada et al. 1998).

Carotid sinus pressure, SNA, and AP were recorded at 1000 Hz using a 16 bit analog‐to‐digital converter (AD16‐16(PCI)EV, Contec, Japan). The sampling program was custom made. Mean values of SNA and AP corresponding to the five CSP levels were calculated by averaging respective signals from the 90th to 100th sec of each step immediately before the arterial blood sampling. In each animal, the SNA value measured after ganglionic blockade was assigned to 0%. The SNA value obtained at the CSP of 60 mmHg under the control condition before desipramine was assigned to 100%. The same normalization factor was applied to describe SNA after desipramine.

A coefficient of variation (CV), which is the ratio of the standard deviation to the mean, was calculated at each CSP level to describe the distribution of the catecholamine data among animals. The relationship between SNA and AP and that between SNA and the absolute value of plasma catecholamine concentration were quantified using linear regression ($y=b_{0}+b_{1}\times x$), where *b*_0_ and *b*_1_ represent the intercept and slope, respectively. A coefficient of determination (*r* ^2^) was calculated to quantify the linear association between the two variables.

### Statistical analysis

Data are presented as mean ± standard error (SE) values except where otherwise stated. Body weight, biventricular weight, and baseline hemodynamics were compared between the NC and MI groups using unpaired *t*‐tests. Changes in absolute values of NE and Epi concentrations in response to an increase in CSP under the baseline condition were examined by a one‐way repeated‐measures analysis of variance (ANOVA) followed by Dunnett's test (Glantz 2002). Overall effects of desipramine on SNA, absolute values of plasma catecholamine concentrations, and AP were examined in each group using a two‐way repeated‐measures ANOVA (the two factors were desipramine and the CSP level). The differences in the intercept and slope of linear regression were examined using the bootstrap method, which does not assume normal distribution to test the difference of a given statistical quantity (Efron and Tibshirani 1994). In all of the statistical analyses, the difference was considered to be significant when *P *<* *0.05. For regression parameters, the Bonferroni correction with a factor of 3 was applied taking into account the nature of multiple comparisons (i.e., the comparison between the NC and MI groups under the baseline condition without desipramine, the comparison before and after desipramine within the NC group, and the comparison before and after desipramine within the MI group); hence, the original *P‐*values less than 0.01/3 and 0.05/3 are reported as *P *<* *0.01 and *P *<* *0.05, respectively.

## Results

**Figure 1:**
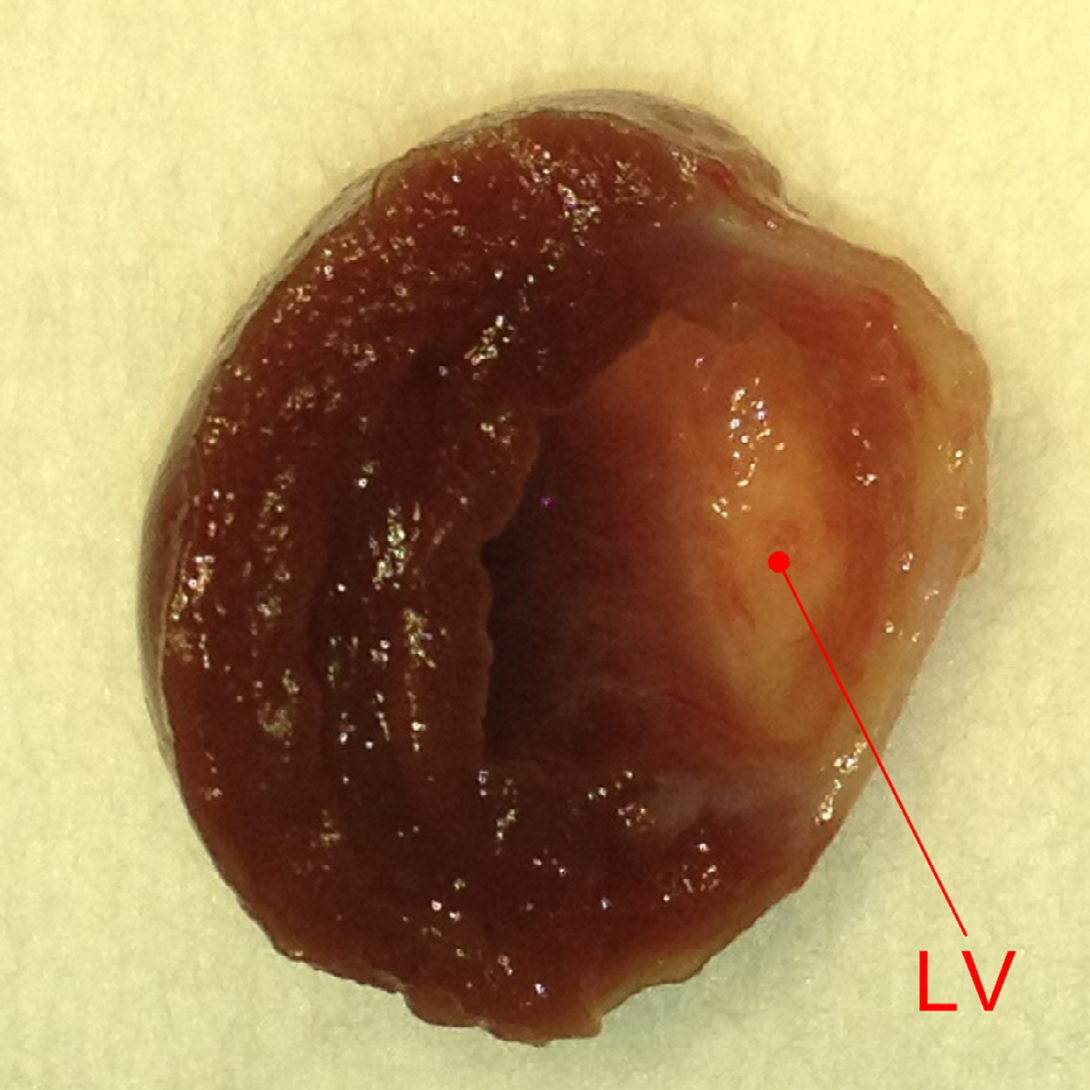
Typical cross section of the heart obtained from a rat with myocardial infarction. The free wall of the left ventricle (LV) turned into a thin membrane‐like scar tissue.

In the MI group, most of the left ventricular free wall turned into thin scar tissue, which was identified by postmortem macroscopic inspection (Fig. 1). Table 1 summarizes age, body weight, biventricular weight, and baseline hemodynamics measured under anesthesia and artificial ventilation but before isolating carotid sinus regions. Age at the time of experiment was not significantly different between the NC and MI groups, but body weight was significantly lower in the MI group. Biventricular weight was heavier in the MI than in the NC group, in both the absolute value and the value normalized to body weight. Central venous pressure tended to be higher in the MI compared with the NC group. Baseline mean AP did not differ significantly between the two groups, and baseline heart rate tended to be lower in the MI than in the NC group.

**Table 1:** Body weight, biventricular weight, and baseline hemodynamics in normal control (NC) and myocardial infarction (MI) groups

	NC (*n* = 6)	MI (*n* = 6)	*P*‐value
Age at the time of experiment, weeks	15.9 ± 0.5	16.8 ± 0.2	0.123
Body weight, g	457.5 ± 13.8	413.3 ± 13.3	0.044
Biventricular weight, g	0.945 ± 0.041	1.135 ± 0.034	0.005
Normalized biventricular weight, g kg^−1^	2.062 ± 0.039	2.751 ± 0.083	<0.001
Central venous pressure, mmHg	1.97 ± 0.09	2.31 ± 0.13	0.052
Mean arterial pressure, mmHg	131.9 ± 3.4	124.6 ± 4.5	0.228
Heart rate, beats per min	432.1 ± 19.6	377.8 ± 16.3	0.059

Values are means ± SE. *P*‐value was calculated by unpaired *t*‐test.

**Figure 2:**
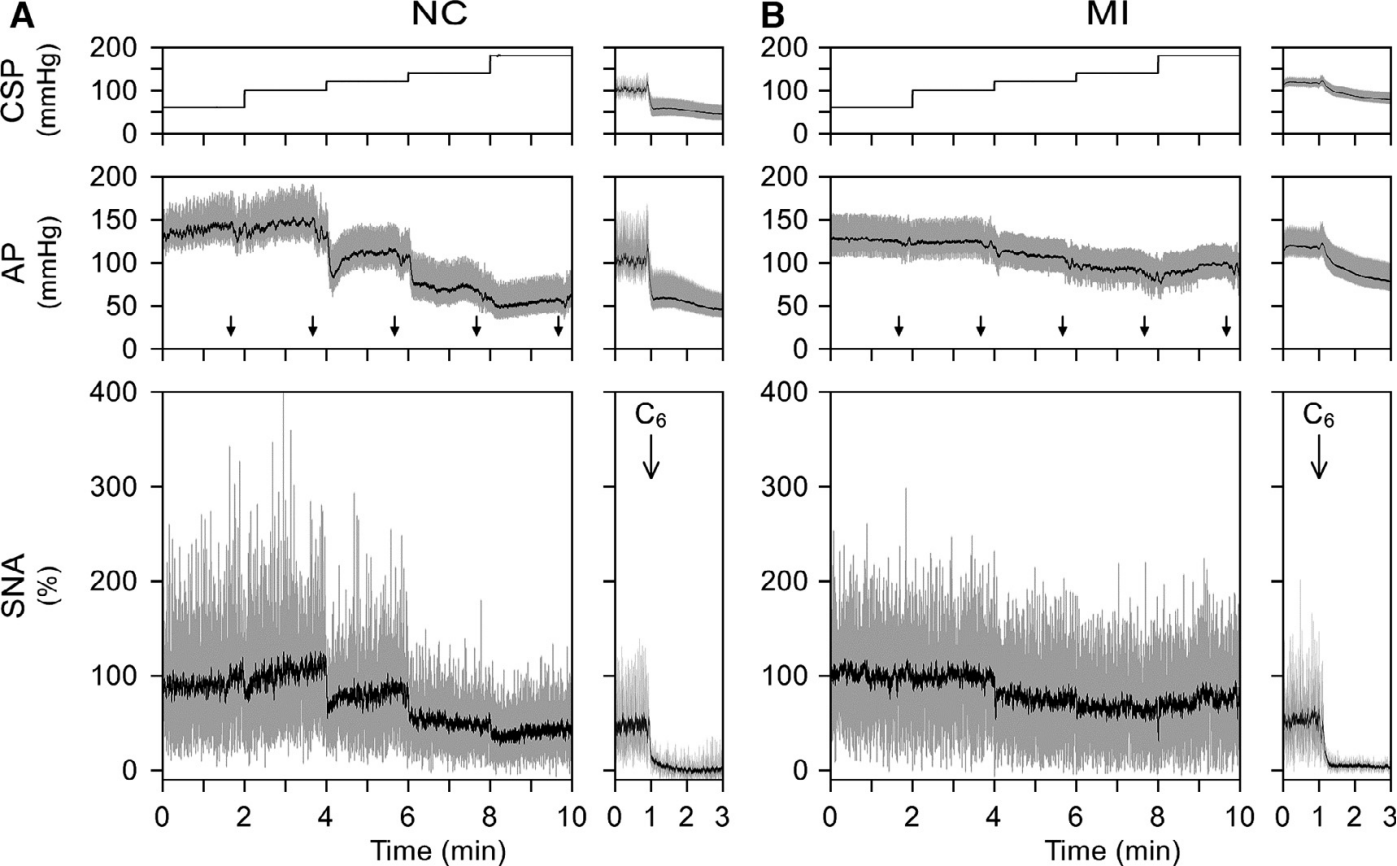
(A and B) Typical time series of carotid sinus pressure (CSP), arterial pressure (AP), and sympathetic nerve activity (SNA) obtained in a normal control (NC) rat and a rat after myocardial infarction (MI), respectively. A stepwise increase in CSP decreased AP and SNA in both rats. The magnitude of AP and SNA responses were, however, smaller in the MI than in the NC rat. The gray and black lines in the AP plot indicate 1000 Hz sampled data and the 2 sec moving averaged signal, respectively. The down arrowheads indicate the timings of blood sampling. The gray and black lines in the SNA plot indicate 10 Hz resampled data and the 2 sec moving averaged signal, respectively. At the end of the experiment, intravenous administration of hexamethonium (C6) reduced SNA to the noise level, which was treated as zero.

Typical time series obtained from NC and MI rats are shown in Figure 2. The gray and black lines in the AP panels represent 1000 Hz sampled data and 2 sec moving averaged signals, respectively. The down arrowheads in the AP panels indicate the timings of blood sampling. The gray and black lines in the SNA panels represent 10 Hz resampled data and 2 sec moving averaged signals, respectively. In both rats, an increase in CSP decreased AP and SNA. The magnitude of AP response was smaller in the MI than in the NC rat. The minimum percent SNA observed during the staircase‐wise CSP input was higher in the MI than in the NC rat, indicating an impairment of baroreflex‐mediated SNA suppression. In both rats, burst activities in SNA disappeared after the administration of hexamethonium (C6) at the end of the experiment.

**Figure 3:**
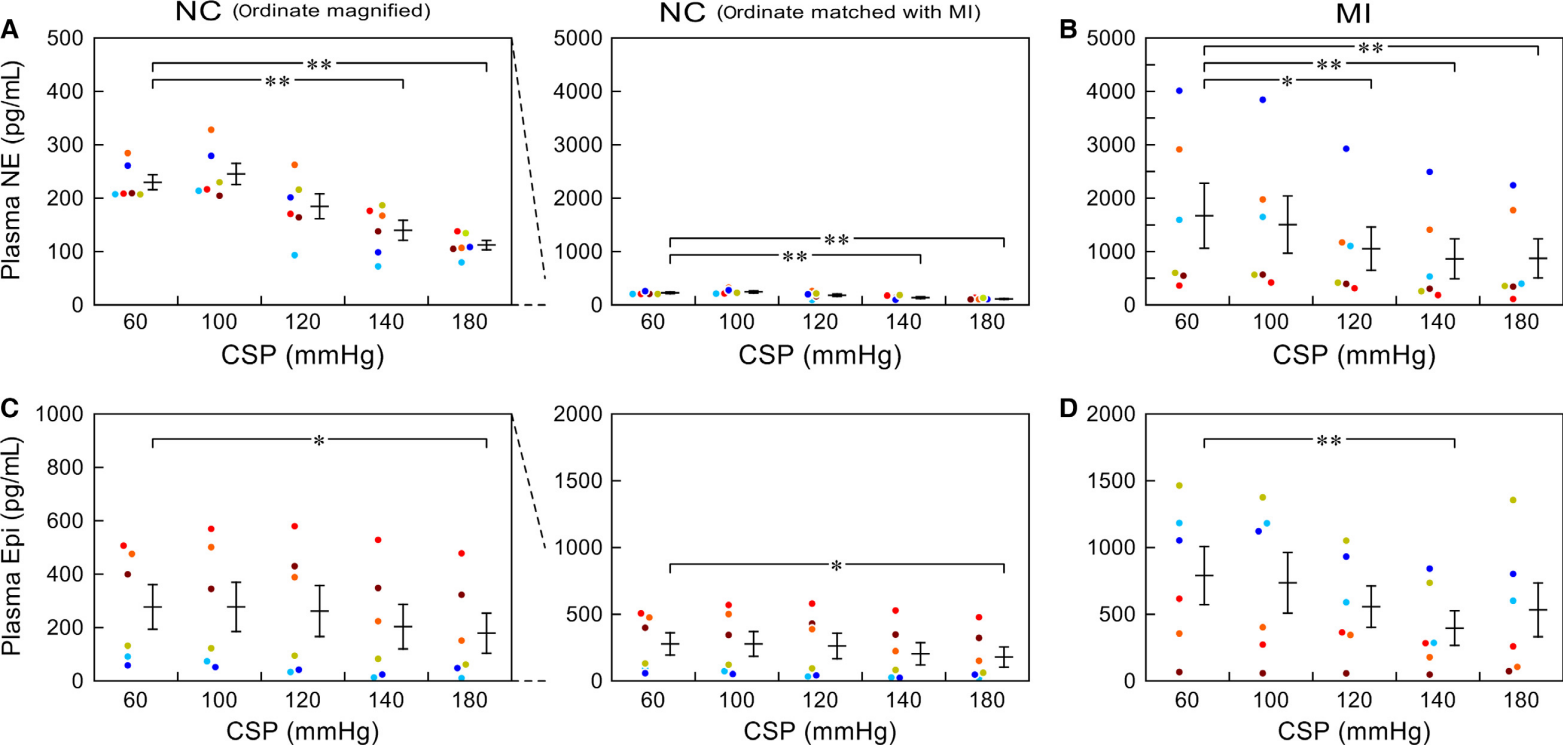
(A and B) Plasma norepinephrine (NE) concentrations obtained in normal control (NC) and myocardial infarction (MI) groups, respectively. (C and D) Plasma epinephrine (Epi) concentrations obtained in NC and MI groups, respectively. Data of individual animals (small dots) and their mean ± SE values are shown. CSP: carotid sinus pressure. *P <* *0.05 and **P <* *0.01 by Dunnett's test. Within each group, the dots in the same color represent data obtained from the same animal.

Figure 3 illustrates plasma catecholamine concentrations in individual animals (small dots) and their mean ± SE values measured at each CSP level obtained in the NC (Fig. 3A and C) and MI (Fig. 3B and D) groups. Within each group, the dots in the same color represent data obtained from the same animal. For plasma NE concentrations in the NC group (Fig. 3A), the ordinate of the left panel is magnified 10 times than that of the right panel. Plasma NE concentrations in the NC group (Fig. 3A) decreased as the CSP increased. Plasma NE concentrations in the MI group (Fig. 3B) were much higher and showed larger variance than those in the NC group, yielding a higher CV in the MI (0.87–1.06) than in the NC (0.15–0.33) group. Nonetheless, the plasma NE concentrations in the MI group also decreased as the CSP increased.

For plasma Epi concentrations in the NC group (Fig. 3C), the ordinate of the left panel is magnified twice than that of the right panel. Plasma Epi concentrations in the NC group (Fig. 3C) were more variable than plasma NE concentrations, showing the CV ranging from 0.74 to 1.03. While the response to an increase in CSP was not obvious in some animals, the mean Epi concentration at the CSP of 180 mmHg was significantly lower than that at the CSP of 60 mmHg in the NC group. Plasma Epi concentrations in the MI group (Fig. 3D) showed the CV ranging from 0.67 to 0.93. While the mean levels of Epi in the MI group were 2–3 times higher than those in the NC group, individual plasma Epi concentrations in some MI rats overlapped with the range of plasma Epi concentrations in the NC group. Plasma Epi concentration at the CSP of 140 mmHg was significantly lower than that at the CSP of 60 mmHg in the MI group.

**Figure 4:**
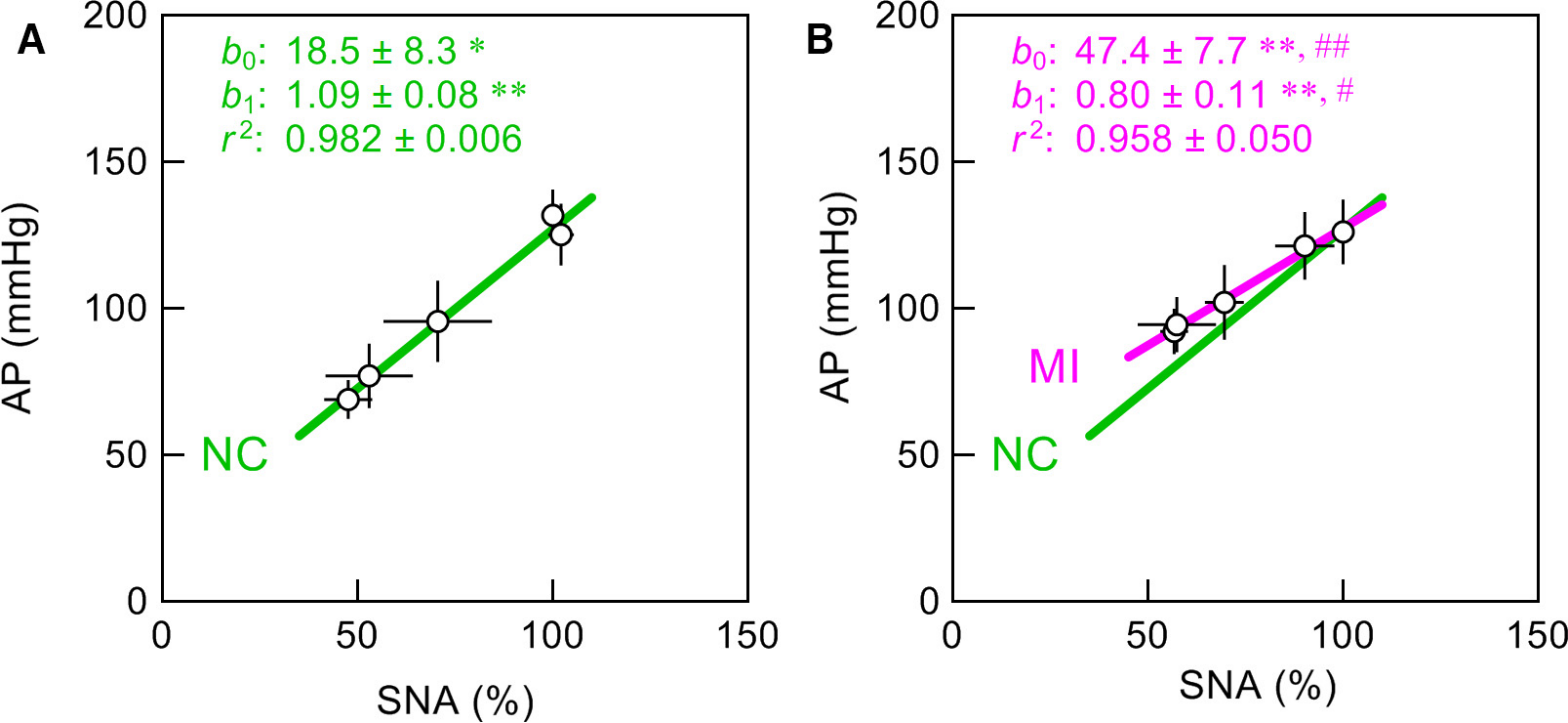
(A and B) Regression lines between sympathetic nerve activity (SNA) and arterial pressure (AP) obtained in normal control (NC) and myocardial infarction (MI) groups, respectively. The green line in panel B duplicates the regression line in panel A for comparison. *b*_0_: intercept, *b*_1_: slope, *r* ^2^: coefficient of determination. Data points are mean ± SE. *P <* *0.05 and **P <* *0.01 indicate that parameter values were significantly different from zero. ^#^ *P *<* *0.05 and ^##^ *P *<* *0.01 indicate that parameter values were significantly different between the NC and MI groups.

The relationship between SNA and AP was approximately linear with a positive slope in both the NC (Fig. 4A) and MI (Fig. 4B) groups. The green line in Figure 4B duplicates the regression line in Figure 4A for comparison. The intercept was significantly higher (*P *<* *0.01) and the slope was significantly lower (*P *<* *0.05) in the MI group compared with the NC group.

**Figure 5:**
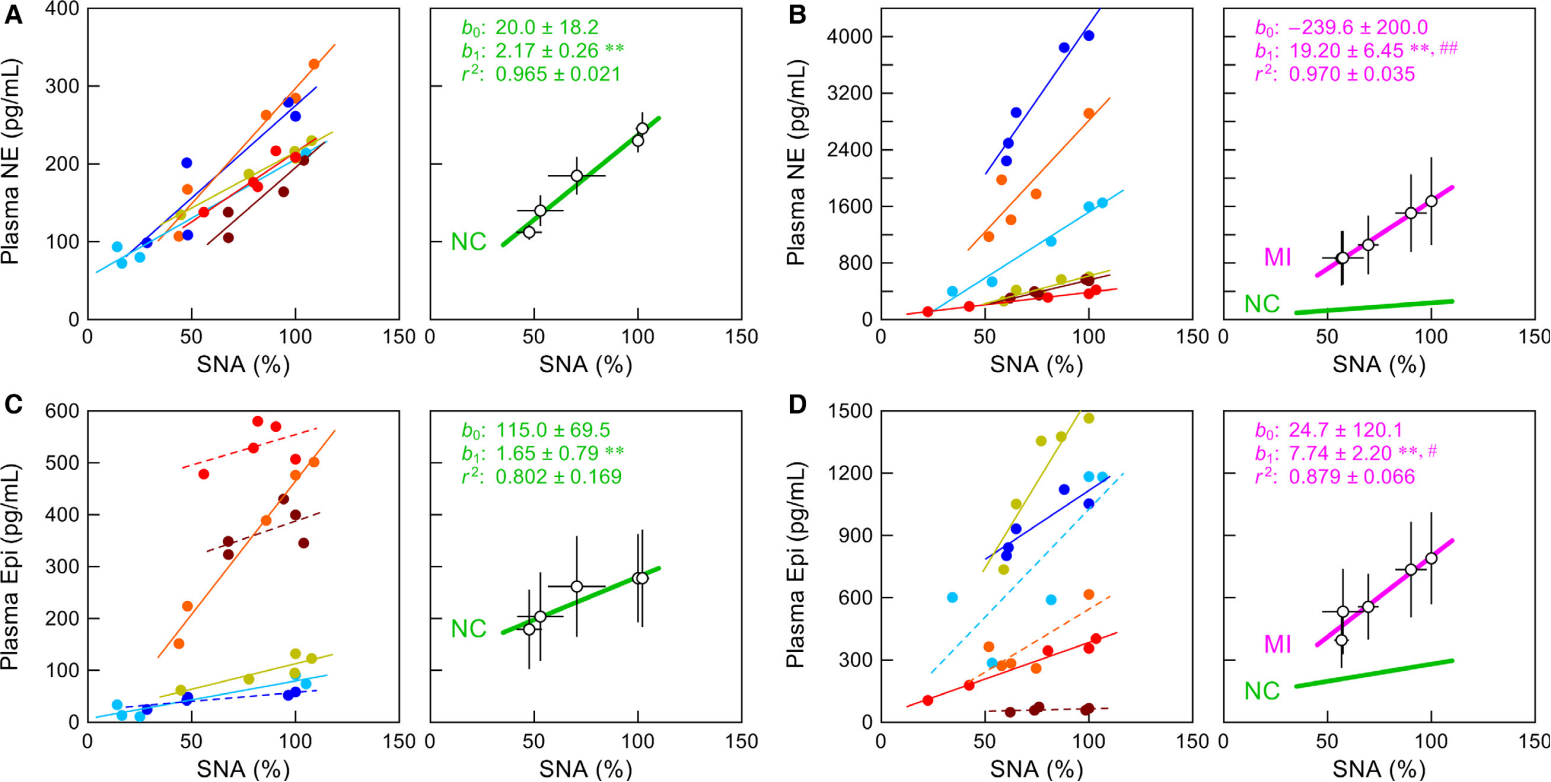
(A and B) Regression lines between sympathetic nerve activity (SNA) and plasma norepinephrine (NE) concentration in individual rats (left) and in group‐averaged data (right) for normal control (NC) and myocardial infarction (MI) groups, respectively. (C and D) Regression lines between SNA and plasma epinephrine (Epi) concentration in individual rats (left) and in group‐averaged data (right) for the NC and MI groups, respectively. The regression line is depicted in solid line when the slope is significantly different from zero (*P *<* *0.05) and in broken line when the slope is not significantly different from zero. Within each group, the regression lines in the same color represent the data obtained from the same animal. The green lines in the right panels of B and D duplicate the regression lines in the right panels of A and C, respectively, for comparison. *b*_0_: intercept, *b*_1_: slope, *r* ^2^: coefficient of determination. Data points are mean ± SE. *P <* *0.05 and **P <* *0.01 indicate that parameter values were significantly different from zero. ^#^ *P *<* *0.05 and ^##^ *P *<* *0.01 indicate that parameter values were significantly different between the NC and MI groups.

The relationship between SNA and plasma NE concentration was approximately linear with a positive slope in individual animals for both the NC (Fig. 5A, left) and MI (Fig. 5B, left) groups. Group‐wise analyses also indicate that the slope was significantly different from zero in both groups (Fig. 5A, right and B, right), and was approximately nine times higher (*P *<* *0.01) in the MI group compared with the NC group.

In the NC group, the slope of the relationship between SNA and plasma Epi concentration was significantly different from zero in three rats (Fig. 5C, left, solid lines) but not in the remaining three rats (Fig. 5C, left, broken lines). When the data from the six rats were analyzed together, the slope was significantly different from zero (Fig. 5C, right). In the MI group, the slope of the relationship between SNA and plasma Epi concentration was significantly different from zero in three rats (Fig. 5D, left, solid lines) but not in the remaining three rats (Fig. 5D, left, broken lines). When the data from the six rats were analyzed together, the slope was significantly different from zero (Fig. 5D, right). Group‐wise analyses indicate that the slope was approximately 4.5 times higher (*P *<* *0.05) in the MI than in the NC group.

**Figure 6:**
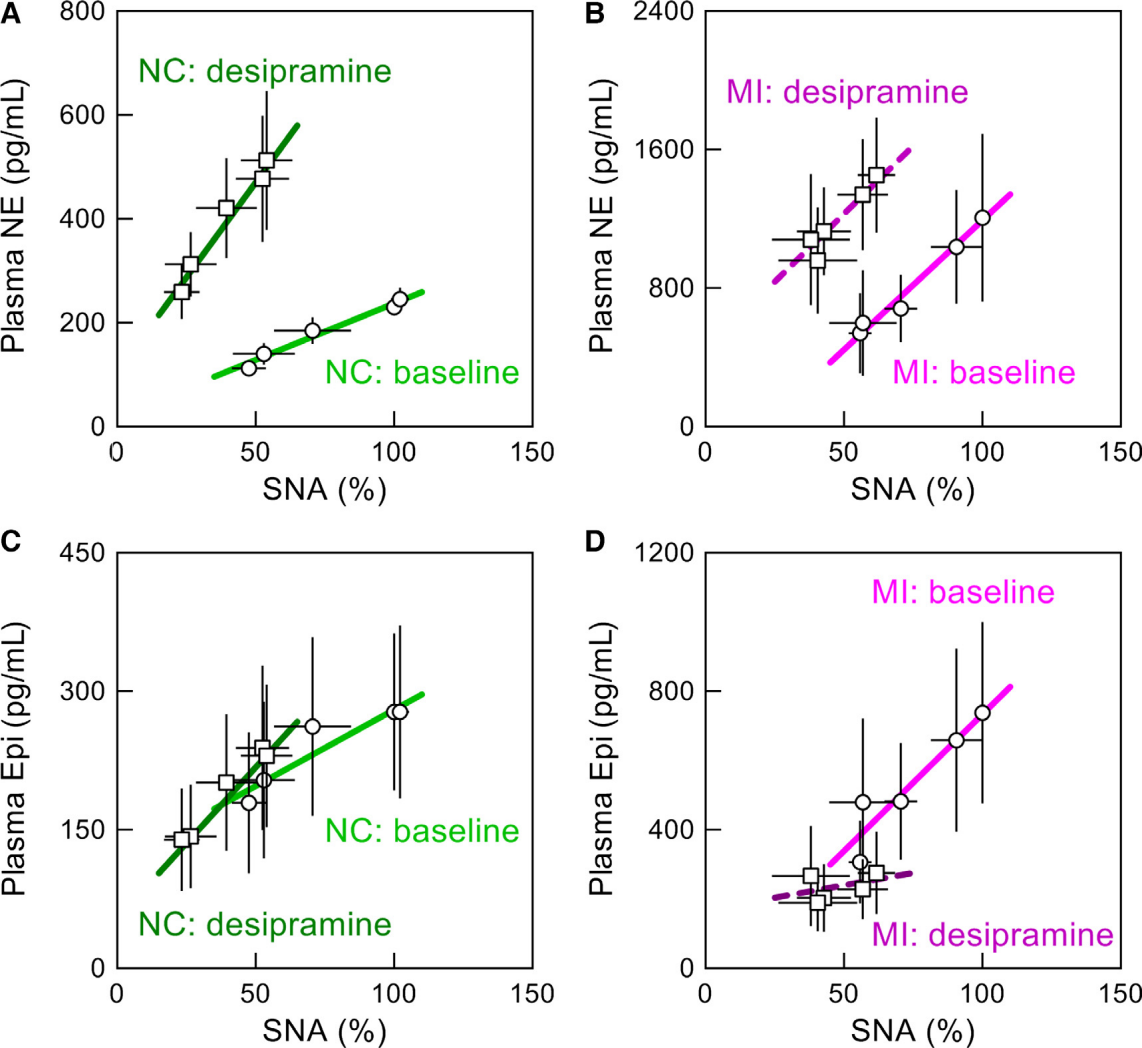
(A and B) Regression lines between sympathetic nerve activity (SNA) and plasma norepinephrine (NE) concentration before (green and purple lines) and after (dark green and dark purple lines) desipramine obtained in normal control (NC) and myocardial infarction (MI) groups, respectively. (C and D) Regression lines between SNA and plasma epinephrine (Epi) concentration obtained before (green and purple lines) and after (dark green and dark purple lines) desipramine in the NC and MI groups, respectively. The regression line is depicted in solid line when the slope is significantly different from zero (*P *<* *0.05) and in broken line when the slope is not significantly different from zero. Data points are mean ± SE. The baseline data in the NC group are the same as those presented in Figure 5A and C. The baseline data in the MI group are different from those presented in Figure 5B and D because of the decreased number of analyzed animals from 6 to 5 for pairwise comparison of the effects of desipramine (see main text for details).

Two‐way repeated‐measures ANOVA within the NC group indicated that desipramine reduced SNA, increased NE, and decreased Epi (Table 2). Desipramine did not significantly affect AP. The effects of CSP on SNA, NE, Epi, and AP were all statistically significant, and there were no interaction effects between desipramine and the CSP level. Shown in Figure 6A are regression lines between SNA and plasma NE concentration before (green line) and after (dark green line) desipramine in the NC group. Desipramine did not change the intercept significantly (from 20.0 ± 18.2 to 105.0 ± 66.1 pg mL^−1^) but increased the slope (from 2.17 ± 0.26 to 7.30 ± 2.04 pg mL^−1^ %^−1^, *P* < 0.01). Figure 6C shows regression lines between SNA and plasma Epi concentration before (green line) and after (dark green line) desipramine in the NC group. Desipramine did not affect the intercept significantly (from 115.0 ± 69.5 to 53.8 ± 80.7 pg mL^−1^) but increased the slope (from 1.65 ± 0.79 to 3.28 ± 1.74 pg mL^−1^ %^−1^, *P *<* *0.05).

**Table 2:** Effects of desipramine (DMI) and carotid sinus pressure (CSP) on sympathetic nerve activity (SNA), plasma norepinephrine (NE) and epinephrine (Epi) concentrations, and arterial pressure (AP) in the normal control group

	Before DMI					After DMI					*P*‐value		
	CSP_60_	CSP_100_	CSP_120_	CSP_140_	CSP_180_	CSP_60_	CSP_100_	CSP_120_	CSP_140_	CSP_180_	DMI	CSP	Interaction
SNA (%)	100	102.1 ± 2.9	70.5 ± 13.5	52.9 ± 10.8	47.5 ± 9.2	52.4 ± 9.2	53.9 ± 8.9	39.4 ± 10.6	26.5 ± 9.0	23.3 ± 6.1	<0.001	<0.001	0.177
NE (pg mL^−1^)	229.9 ± 14.0	245.5 ± 19.8	184.8 ± 23.3	140.1 ± 18.7	112.3 ± 8.8	477.0 ± 119.6	512.5 ± 131.6	420.7 ± 94.1	312.4 ± 60.1	259.1 ± 50.0	<0.001	0.001	0.718
Epi (pg mL^−1^)	277.6 ± 83.8	277.7 ± 92.5	261.8 ± 95.6	203.8 ± 83.6	179.2 ± 75.3	238.6 ± 87.9	230.1 ± 76.0	201.1 ± 72.8	142.9 ± 54.9	139.3 ± 54.4	0.011	0.003	0.992
AP (mmHg)	131.8 ± 8.3	125.2 ± 10.0	95.6 ± 13.4	77.0 ± 10.5	68.9 ± 6.2	132.9 ± 10.3	120.6 ± 12.0	96.2 ± 14.4	74.4 ± 8.4	66.4 ± 5.2	0.665	<0.001	0.986

Values are means ± SE, *n* = 6. *P*‐value was calculated by two‐way repeated measures analysis of variance. One factor is DMI (two levels) and the other factor is CSP (five levels). CSP_*x*_ indicates a CSP of *x* mmHg.

One animal in the MI group died during the desipramine protocol, and the following analyses were performed on the remaining five animals. Two‐way repeated‐measures ANOVA within the MI group indicated that desipramine reduced SNA, increased NE, and decreased Epi (Table 3). Desipramine did not significantly affect AP. The effects of CSP were statistically significant on SNA, NE, and AP but not on Epi. There were no interaction effects between desipramine and the CSP level. Figure 6B shows regression lines between SNA and plasma NE concentration before (purple line) and after (dark purple line) desipramine in the MI group. Desipramine did not change the slope significantly (from 14.94 ± 5.80 to 15.69 ± 6.60 pg mL^−1^ %^−1^) but increased the intercept (from −302.0 ± 202.7 to 445.1 ± 270.2 pg mL^−1^, *P* < 0.01). Figure 6D shows regression lines between SNA and plasma Epi concentration in the MI group. While the regression lines drawn over the mean parameter values appeared to be different, the effects of desipramine on the intercept (from −55.9 ± 112.8 to 168.2 ± 155.8 pg mL^−1^) and slope (from 7.90 ± 2.59 to 1.43 ± 1.75 pg mL^−1^ %^−1^) were not statistically significant. In the MI group, the slope of the regression line was significantly different from zero before desipramine (depicted in solid lines) but not after desipramine (depicted in broken lines) for both plasma NE and Epi concentrations.

**Table 3:** Effects of desipramine (DMI) and carotid sinus pressure (CSP) on sympathetic nerve activity (SNA), plasma norepinephrine (NE) and epinephrine (Epi) concentrations, and arterial pressure (AP) in the myocardial infarction group

	Before DMI					After DMI					*P*‐value		
	CSP_60_	CSP_100_	CSP_120_	CSP_140_	CSP_180_	CSP_60_	CSP_100_	CSP_120_	CSP_140_	CSP_180_	DMI	CSP	Interaction
SNA (%)	100	90.6 ± 8.8	70.5 ± 5.5	55.8 ± 3.8	56.8 ± 11.8	56.7 ± 8.8	61.7 ± 6.4	42.7 ± 9.4	40.5 ± 13.9	38.0 ± 13.7	<0.001	<0.001	0.470
NE (pg mL^−1^)	1206.2 ± 478.3	1038.0 ± 322.4	682.0 ± 188.3	539.8 ± 225.7	598.8 ± 298.8	1339.4 ± 315.2	1452.6 ± 326.3	1127.7 ± 247.8	959.6 ± 301.4	1079.1 ± 372.1	<0.001	0.006	0.832
Epi (pg mL^−1^)	737.8 ± 258.7	658.6 ± 261.1	481.8 ± 165.7	306.7 ± 115.8	479.4 ± 238.3	228.3 ± 83.6	275.5 ± 115.8	203.1 ± 94.9	189.3 ± 79.2	266.7 ± 141.5	<0.001	0.093	0.272
AP (mmHg)	136.1 ± 3.9	131.7 ± 4.5	110.8 ± 10.4	99.1 ± 2.5	99.1 ± 9.3	128.9 ± 5.0	128.0 ± 6.0	104.5 ± 10.2	90.4 ± 7.8	99.9 ± 12.7	0.274	<0.001	0.969

Values are means ± SE, *n* = 5. *P*‐value was calculated by two‐way repeated measures analysis of variance. One factor is DMI (two levels) and the other factor is CSP (five levels). CSP_*x*_ indicates a CSP of *x* mmHg.

## Discussion

We have shown that the relationship between SNA and plasma NE concentration during acute baroreflex‐mediated changes was approximately linear with a positive slope for both NC and MI groups. The slope of the regression line was much higher in the MI than in the NC group. Plasma Epi concentration also positively correlated with SNA by group‐wise analyses, but half of the animals in each group did not show significant regression (i.e., the slope of the regression line was not significantly different from zero). Neuronal NE uptake blockade by intravenous desipramine reduced SNA, increased plasma NE concentration, and decreased plasma Epi concentration in both the NC and MI groups.

### A chronic rat model of MI

It may be pertinent to briefly discuss the chronic rat model of MI used in this study. A previous study indicates that left ventricular weight relative to body weight decreases after a left coronary occlusion in the phase of scar formation, and recovered to the preocclusion value by 106 days after MI due to compensatory hypertrophy of remaining myocardium (Pfeffer et al. 1991). On the other hand, right ventricular weight relative to body weight increases by 106 days after MI only in rats with large MI (Pfeffer et al. 1991). As a result, biventricular weight relative to body weight significantly increases only in rats with large MI. We have also learned from our past studies that normalized biventricular weight significantly increases only in rats with large MI (Li et al. 2004, 2014). In this study, all rats in the MI group showed normalized biventricular weight greater than 2.5 g kg^−1^ by postmortem examination, as this value was used as a criterion for the successful creation of large MI. Our previous study has indicated that left ventricular function is significantly depressed in rats that underwent a similar left coronary occlusion procedure by the same operator (M.L.) (Li et al. 2004).

Baseline AP was not lower in the MI than in the NC group in this study (Table 1), suggesting that the circulation was still compensated by sympathoexcitation and possible fluid retention. A canine model of pacing‐induced heart failure also indicates that mean AP can be maintained even when cardiac output is significantly decreased (Wang et al. 1990). Artificial ventilation with oxygenated room air during baseline hemodynamic measurements might have also contributed to the maintenance of AP in the MI group.

### Relationships between SNA and AP

We have used the term “peripheral arc” to refer to the input–output relationship between SNA and AP obtained by open‐loop analysis of the arterial baroreflex system (Ikeda et al. 1996; Kawada and Sugimachi 2016). The peripheral arc showed approximately positive linear relationship in both the NC and MI groups, but the slope was significantly lower in the MI than in the NC group (Fig. 4). The underlying mechanisms for the reduced slope of the peripheral arc in a chronic model of MI have been discussed in detail previously (Kawada et al. 2010, 2014b). A potential pitfall in interpreting the peripheral arc is that the slope can depend on how SNA is expressed. If SNA is expressed in absolute amplitude or frequency, rather than percentage in each animal, the maximum SNA is likely to be higher in the MI than in the NC group. This means that the slope of the peripheral arc could be much lower in the MI group relative to the NC group if SNA is expressed in absolute units (Kawada et al. 2010). The same is true for the comparison of the slope in the SNA–NE relationship between the NC and MI groups. In the following section, a possibility is discussed as to calibrating SNA between the NC and MI groups by taking advantage of the linearity of the relationship between SNA and endogenous plasma NE concentration, though there is also room for argument about to what extent plasma NE concentration accurately reflects the magnitude of SNA to end organs.

### Relationship between SNA and plasma NE concentration

Our previous study has demonstrated that plasma NE concentration changes almost linearly with SNA during a staircase‐wise CSP input in Wistar‐Kyoto rats (Kawada et al. 2014a). As we have abundant experience and data regarding a chronic model of MI in Sprague–Dawley rats (Li et al. 2004, 2014; Kawada et al. 2010, 2014b, 2015), we thought it was necessary to provide control data in Sprague–Dawley rats rather than Wistar‐Kyoto rats. We confirmed that the SNA–NE relationship was approximately linear with a positive slope in the NC group using Sprague–Dawley rats (Fig. 5A). The slope was higher than that obtained previously in Wistar‐Kyoto rats (0.957 ± 0.090 pg mL^−1^ %^−1^) (Kawada et al. 2014a), further suggesting a difference in the baroreflex‐mediated sympathetic control between the two normotensive strains of rats (Turner et al. 2015b).

This study extended our previous findings in that the positive correlation between SNA and plasma NE concentration was also observed in the MI group despite the large interindividual variance of plasma NE concentration (Fig. 5B). Since plasma NE concentration is measured in absolute units (pg mL^−1^), the approximate linear relationship may allow us to calibrate SNA by endogenous plasma NE concentration for comparison between the NC and MI groups. It should be kept in mind, however, that plasma NE concentrations have several shortcomings that limit their utility in assessing the magnitude of SNA to end organs (Floras 2003). Plasma NE concentration depends not only on the release of NE but also on the factors for NE removal that operate between the synapse and the circulation (Goldstein et al. 1983). Hence, a high plasma NE level does not necessarily indicate a high level of SNA to end organs (Goldstein et al. 2003).

### Effects of neuronal NE uptake blockade

While an increased sympathetic outflow from the central nervous system most likely contributed to the high levels of plasma NE concentration in chronic heart failure, peripheral modulation of NE release and disposition can occur in diseased conditions via several mechanisms: some of which are related to neuronal NE uptake as discussed below. When NE is released via nonexocytotic mechanism in ischemic myocardium, myocardial interstitial NE concentration can increase more than 100 times the baseline level independent of SNA (Kawada et al. 2000; Akiyama and Yamazaki 2001). In contrast, the present results indicate that the high levels of plasma NE in the MI group were still under the influence of SNA (even in the individual with the highest plasma NE concentration, Fig. 5B, blue), which may not be consistent with the SNA‐independent nonexocytotic release mechanism. The nonexocytotic release is mediated by reverse transport via the neuronal NE uptake transporter. Hence, neuronal NE uptake blockade by desipramine reduces the nonexocytotic NE release (Schömig et al. 1987; Akiyama and Yamazaki 2001). In contrast, desipramine did not reduce but rather increased plasma NE concentration in the MI group (Fig. 6B), which also suggests that the nonexocytotic NE release did not play a significant role in producing the high levels of plasma NE in this group.

Since a large part of NE released into the synaptic cleft is removed by neuronal NE uptake mechanism, the impairment of neuronal NE uptake increases the level of NE at the synaptic cleft, and resultantly increases the diffusion of NE into the bloodstream. The slope of the regression line between SNA and plasma NE concentration became steeper after neuronal NE uptake blockade by desipramine in the NC group (Fig. 6A), which is in agreement with our previous result in Wistar‐Kyoto rats (Kawada et al. 2014a). Neuronal NE uptake is driven by energy drawn from the sodium gradient across the plasma membrane (Schwartz 2000), which is maintained by sodium extrusion through Na^+^, K^+^‐ATPase. The Na^+^, K^+^‐ATPase activity may be reduced in heart failure (Despa and Bers 2013), leading to dysfunction of the neuronal NE uptake mechanism. Actually, decreased efficiency of cardiac neuronal NE uptake has been reported in patients with congestive heart failure (Eisenhofer et al. 1996). Abnormality in neuronal NE uptake is also observed in small arteries obtained by gluteal biopsies in patients with chronic heart failure, where neuronal NE uptake blockade by cocaine does not affect NE‐induced vasoconstriction in these vessels (Hillier et al. 1999). If the impairment of neuronal NE uptake serves as the chief mechanism for an increased plasma NE concentration in the MI group, neuronal NE uptake blockade may not further affect plasma NE concentration as in the case with the biopsied small arteries. Contrary to this hypothesis, desipramine increased plasma NE concentration in the MI group (Fig. 6B). Therefore, it is likely that neuronal NE uptake at the sympathetic nerve terminals throughout the body, except for possibly at the cardiac sympathetic nerve, had been operating effectively before desipramine administration, which limited the diffusion of NE into the bloodstream. In contrast to the NC group, the intercept rather than the slope was changed by desipramine in the MI group. Whether this change has specific pathological meaning remains inconclusive due to the large variance of plasma NE concentration in the MI group.

Aside from neuronal NE uptake, other peripheral mechanisms could cause a disproportional increase in plasma NE concentration relative to SNA. Angiotensin II, which is known to be elevated in chronic heart failure, can affect ganglionic transmission (Ma et al. 2004) and facilitate NE release from sympathetic nerve terminals (Reid 1992). An increased level of plasma Epi can act on presynaptic *β*_2_‐adrenergic receptors and potentiate NE release from sympathetic nerve terminals (Majewski et al. 1981; Floras 1992). Furthermore, the autoinhibition of NE release via presynaptic *α*_2_‐adrenergic receptors is impaired in heart failure (Aggarwal et al. 2001). The effects of such peripheral modulations need to be taken into consideration if we use plasma NE concentration as a surrogate for SNA.

### Relationship between SNA and plasma Epi concentration

In this study, blood samples were taken from the aorta, and thus, catecholamines came from both the adrenal gland and from neurotransmitter spillover from all organs including systemic vasculature. Plasma Epi is not detectable after bilateral adrenalectomy (Péronnet et al. 1994), indicating that the primary source of plasma Epi is the adrenal gland. On the other hand, bilateral adrenalectomy does not affect basal plasma NE concentration (Péronnet et al. 1994), suggesting that only a small amount of plasma NE comes from the adrenal gland under resting conditions (Goldstein et al. 2003). Epinephrine is different from NE in that it is directly released into the bloodstream from the adrenal medulla (Goldstein et al. 2003). While plasma Epi concentration is positively correlated with SNA by group‐wise analyses (Fig. 5C, right), some rats did not show significant regression (Fig. 5C, left), suggesting that plasma NE concentration may reflect SNA better than plasma Epi concentration. Since we recorded SNA from a postganglionic branch of the splanchnic nerve, it may not conform to the preganglionic activity directed to the adrenal gland. When preganglionic discharge is assessed by the dialysate acetylcholine levels in the adrenal medulla, dialysate Epi concentration positively correlates with the dialysate acetylcholine concentration (Akiyama et al. 2004). Dissociation between plasma Epi and NE concentrations occurs depending on different types of stressors (Goldstein et al. 1983; Young et al. 1984; Kvetňanský et al. 1998).

The slope of the relationship between SNA and plasma Epi concentration was higher in the MI than in the NC group by group‐wise analyses. Possible dysfunction of neuronal NE uptake does not account for the increased Epi concentration in the MI group because Epi is a poorer substrate than NE for the neuronal NE uptake transporter (Goldstein et al. 2003). Intravenous desipramine acts centrally to reduce SNA (Svensson and Usdin 1978; Eisenhofer et al. 1991; Kawada et al. 2004). After desipramine, plasma Epi concentration decreased along with SNA, whereas plasma NE concentration increased, confirming that Epi is a poorer substrate than NE for the neuronal NE uptake transporter.

### Relationship between CSP and SNA

While an increase in CSP generally reduced SNA, there was a paradoxical increase in SNA when CSP was stepped from 60 to 100 mmHg in the NC rat (Fig. 2A). This change conformed to the change in the plasma NE concentration in the NC group (Fig. 3A). The phenomenon can be partly explained by the fact that the relationship between baroreceptor activity and input pressure is not monotonous near the threshold input pressure (Bolter et al. 2011). A similar phenomenon has been also observed during a staircase‐wise CSP input in Wistar‐Kyoto rats and spontaneously hypertensive rats (Sata et al. 2015), and is more exaggerated in spontaneously hypertensive rats.

In the MI group, SNA and plasma catecholamine concentrations were not necessarily lower at a CSP of 180 mmHg than at a CSP of 140 mmHg (Fig. 2B, Tables 3). While it was not mentioned, a similar phenomenon can be observed in our previous study using rats survived 100–200 days after MI (Fig. 4a in reference Kawada et al. 1998). The sustained suppression of SNA at high CSP levels is mediated by unmyelinated C‐fibers in baroreceptor afferents (Turner et al. 2015a,b). Aside from general sympathetic activation due to central mechanism (Liu and Zucker 1999; Leenen 2007), we speculate that systemic inflammation associated with chronic heart failure may damage C‐fibers relative to A‐fibers, impairing the baroreflex‐mediated SNA suppression at high CSP levels.

In our previous studies, the CSP–SNA relationship was quantified using four‐parameter logistic function (Kent et al. 1972) based on data obtained at seven different CSP levels (Kawada et al. 2010, 2014b), which revealed a reduction in the response range of SNA (i.e., the difference between the maximum and minimum SNA during the staircase‐wise CSP input) in MI rats. In this study, however, we did not quantify the CSP–SNA relationship in detail because the number of data points (five different CSP levels) may not be sufficient to estimate the four parameters with reasonable accuracy.

### Clinical implication

Chronic heart failure is partly characterized by autonomic abnormality: excess sympathetic activation and vagal withdrawal. While high sympathetic tone may be necessary to maintain blood perfusion to end organs during the acute phase of heart failure, sustained sympathetic excitation exerts a significant burden on the failing heart and aggravates the disease condition. To suppress excess sympathetic effects, pharmacological agents such as *β*‐blockers (Foody et al. 2002), angiotensin‐converting enzyme inhibitors (Flather et al. 2000), and angiotensin II receptor blockers (Pitt et al. 2000), are used for therapy of heart failure. Nevertheless, the mortality rate of chronic heart failure remains high, and additional therapeutic strategies need to be developed (Li et al. 2004, 2014).

The arterial baroreflex system is one of the most important and powerful negative feedback systems that regulates AP via control of SNA. While the long‐term ability of the arterial baroreflex to control a mean level of AP has been somewhat disregarded (Cowley et al. 1973), recent studies indicate an involvement of the arterial baroreflex in long‐term AP regulation (Thrasher 2002; Lohmeier et al. 2010). Based on such results, investigators have regained interest in baroreflex activation therapy (BAT), which is being explored for the treatment of drug‐resistant hypertension (Bakris et al. 2012; Victor 2015). BAT is also being examined as a possible treatment for patients with chronic heart failure (Abraham et al. 2015). In this study, plasma NE concentration was reduced, by a considerable degree, by activating the carotid sinus baroreflex in the MI group (Fig. 3B), despite a high baseline level of plasma NE. While carotid sinus baroreflex function is depressed in heart failure (White 1981; Wang et al. 1990; Kawada et al. 2010) and the effects of BAT may be interrupted to a certain extent via sympathoexcitatory mechanisms within the central nervous system (Liu and Zucker 1999; Leenen 2007), the present results may provide additional rationale for the sympathetic suppression via the activation of the arterial baroreflex in chronic heart failure.

### Limitations

First, since it is not determined to what extent the anesthesia affected plasma catecholamine concentrations, the results need to be carefully interpreted when extrapolating them to understand sympathetic cardiovascular regulation under conscious conditions. Furthermore, since anesthetized animals were artificially ventilated and we waited for at least 60 min after finishing surgical preparation to reduce surgical effects, respiratory insufficiency in the MI group might have been, in a sense, treated during this stabilization period. Second, we were not able to assess the regional differences in SNA in producing changes in plasma NE (Goldstein et al. 1983) because we only measured SNA from a postganglionic branch of the splanchnic sympathetic nerve. A relatively high *r* ^2^ value in the relationship between SNA and plasma NE concentration, however, indicates that splanchnic SNA may convey a signal common to the systemic sympathetic system as well as a regional activity. Third, the vagi were sectioned to obtain an open‐loop condition for the carotid sinus baroreflex. Hence, any autonomic abnormality associated with the vagal system, with respect to both the afferent and efferent pathways, was not evaluated in this study. Fourth, since we did not perform sham operation in the NC group, the effect of surgery cannot be ruled out in interpreting the data in the MI group. Regarding this limitation, one rat which underwent a left coronary occlusion did not develop significant biventricular remodeling (body weight = 496 g and normalized biventricular weight = 2.27 g kg^−1^) and was excluded from the study. The rat showed near normal values of baseline hemodynamics (central venous pressure = 2.03 mmHg, AP = 126.6 mmHg, and HR = 427.7 beats per min) and did not show elevated plasma NE concentration (the maximum plasma NE concentration was 139.6 pg mL^−1^ at a CSP of 60 mmHg). Hence, we think the elevation of plasma NE concentration observed in the MI group is primarily attributable to the creation of large MI and not to the prolonged effect of past surgery.

## Conclusion

There was an approximately positive linear relationship between SNA and plasma NE concentration in the MI group despite the high levels of plasma NE. While high levels of plasma NE do not necessarily reflect the severity of heart failure (Viquerat et al. 1985) or indicate an increased SNA to end organs (Floras 2003; Goldstein et al. 2003), they may be able to reflect changes in SNA during acute baroreflex‐mediated changes within individual subjects. The positive correlation between SNA and plasma catecholamine levels in the MI group may provide additional rationale for applying BAT in patients with chronic heart failure.

## Conflict of Interest

None declared.

## References

[phy212880-bib-0001] Abraham, W. T. , M. R. Zile , F. A. Weaver , C. Butter , A. Ducharme , M. Halbach , et al. 2015 Baroreflex activation therapy for the treatment of heart failure with a reduced ejection fraction. J. Am. Coll. Cardiol. HF 3:487–496. 10.1016/j.jchf.2015.02.006 25982108

[phy212880-bib-0002] Aggarwal, A. , M. D. Esler , F. Socratous , and D. M. Kaye . 2001 Evidence for functional presynaptic alpha‐2 adrenoceptors and their down‐regulation in human heart failure. J. Am. Coll. Cardiol. 37:1246–1251. 11300430 10.1016/s0735-1097(01)01121-4

[phy212880-bib-0003] Akiyama, T. , and T. Yamazaki . 2001 Myocardial interstitial norepinephrine and dihydroxyphenylglycol levels during ischemia and reperfusion. Cardiovasc. Res. 49:78–85. 11121798 10.1016/s0008-6363(00)00219-4

[phy212880-bib-0004] Akiyama, T. , T. Yamazaki , H. Mori , and K. Sunagawa . 2004 Simultaneous monitoring of acetylcholine and catecholamine release in the in vivo rat adrenal medulla. Neurochem. Int. 44:497–503. 15209418 10.1016/j.neuint.2003.09.001

[phy212880-bib-0005] Bakris, G. L. , M. K. Nadim , H. Haller , E. G. Lovett , J. E. Schafer , and J. D. Bisognano . 2012 Baroreflex activation therapy provides durable benefit in patients with resistant hypertension: results of long‐term follow‐up in the Rheos Pivotal Trial. J. Am. Soc. Hypertens. 6:151–158. 10.1016/j.jash.2012.01.003 22341199

[phy212880-bib-0006] Bolter, C. P. , M. J. Turner , and C. J. Barrett . 2011 The relationship between aortic baroreceptor activity and arterial pressure is not monotonic. J. Physiol. Sci. 61:151–160. 21240644 10.1007/s12576-011-0132-4 PMC10717601

[phy212880-bib-0007] Cowley, A. W. Jr , J. F. Liard , and A. C. Guyton . 1973 Role of baroreceptor reflex in daily control of arterial blood pressure and other variables in dogs. Circ. Res. 32:564–576. 4713198 10.1161/01.res.32.5.564

[phy212880-bib-0008] Despa, S. , and D. M. Bers . 2013 Na^+^ transport in the normal and failing heart – remember the balance. J. Mol. Cell. Cardiol. 61:2–10. 23608603 10.1016/j.yjmcc.2013.04.011 PMC3720717

[phy212880-bib-0009] Efron, B. , and R. J. Tibshirani . 1994 An introduction to the Bootstrap. Chapman & Hall/CRC, New York.

[phy212880-bib-0010] Eisenhofer, G. 2001 The role of neuronal and extraneuronal plasma membrane transporters in the inactivation of peripheral catecholamines. Pharmacol. Ther. 91:35–62. 11707293 10.1016/s0163-7258(01)00144-9

[phy212880-bib-0011] Eisenhofer, G. , T. Saigusa , M. D. Esler , H. S. Cox , J. A. Angus , and P. K. Dorward . 1991 Central sympathoinhibition and peripheral neuronal uptake blockade after desipramine in rabbits. Am. J. Physiol. 260:R824–R832. 1672797 10.1152/ajpregu.1991.260.4.R824

[phy212880-bib-0012] Eisenhofer, G. , P. Friberg , B. Rundqvist , A. A. Quyyumi , G. Lambert , D. M. Kaye , et al. 1996 Cardiac sympathetic nerve function in congestive heart failure. Circulation 93:1667–1676. 8653872 10.1161/01.cir.93.9.1667

[phy212880-bib-0013] Flather, M. D. , S. Yusuf , L. Køber , M. Pfeffer , A. Hall , G. Murray , et al. 2000 Long‐term ACE‐inhibitor therapy in patients with heart failure or left‐ventricular dysfunction: a systematic overview of data from individual patients, ACE‐Inhibitor Myocardial Infarction Collaborative Group. Lancet 355:1575–1581. 10821360 10.1016/s0140-6736(00)02212-1

[phy212880-bib-0014] Floras, J. S. 1992 Epinephrine and the genesis of hypertension. Hypertension 19:1–18. 1309718 10.1161/01.hyp.19.1.1

[phy212880-bib-0015] Floras, J. S. 2003 Sympathetic activation in human heart failure: diverse mechanisms, therapeutic opportunities. Acta Physiol. Scand. 177:391–398. 12609011 10.1046/j.1365-201X.2003.01087.x

[phy212880-bib-0016] Foody, J. M. , M. H. Farrell , and H. M. Krumholz . 2002 *β*‐Blocker therapy in heart failure: scientific review. JAMA 287:883–889. 11851582 10.1001/jama.287.7.883

[phy212880-bib-0017] Glantz, S. A. 2002 Primer of Biostatistics. 5th ed. McGraw‐Hill, New York.

[phy212880-bib-0018] Goldstein, D. S. , R. McCarty , R. J. Polinsky , and I. J. Kopin . 1983 Relationship between plasma norepinephrine and sympathetic neural activity. Hypertension 5:552–559. 6345364 10.1161/01.hyp.5.4.552

[phy212880-bib-0019] Goldstein, D. S. , G. Eisenhofer , and I. J. Kopin . 2003 Sources and significance of plasma levels of catechols and their metabolites in humans. J. Pharmacol. Exp. Ther. 305:800–811. 12649306 10.1124/jpet.103.049270

[phy212880-bib-0020] Hillier, C. , P. J. Cowburn , J. J. Morton , H. J. Dargie , J. G. Cleland , J. J. McMurray , et al. 1999 Structural and functional assessment of small arteries in patients with chronic heart failure. Clin. Sci. (Lond.) 97:671–679. 10585894

[phy212880-bib-0021] Ikeda, Y. , T. Kawada , M. Sugimachi , O. Kawaguchi , T. Shishido , T. Sato , et al. 1996 Neural arc of baroreflex optimizes dynamic pressure regulation in achieving both stability and quickness. Am. J. Physiol. 271:H882–H890. 8853321 10.1152/ajpheart.1996.271.3.H882

[phy212880-bib-0022] Kawada, T. , and M. Sugimachi . 2016 Open‐loop static and dynamic characteristics of the arterial baroreflex system in rabbits and rats. J. Physiol. Sci. 66:15–41. 26541155 10.1007/s12576-015-0412-5 PMC4742515

[phy212880-bib-0023] Kawada, T. , T. Yamazaki , T. Akiyama , T. Sato , T. Shishido , M. Sugimachi , et al. 1998 Liquid chromatographic determination of myocardial interstitial epinephrine. J. Chromatogr. B Biomed. Sci. Appl. 714:375–378. 9766879 10.1016/s0378-4347(98)00221-7

[phy212880-bib-0024] Kawada, T. , T. Yamazaki , T. Akiyama , T. Sato , T. Shishido , M. Inagaki , et al. 2000 Differential acetylcholine release mechanisms in the ischemic and non‐ischemic myocardium. J. Mol. Cell. Cardiol. 32:405–414. 10731440 10.1006/jmcc.1999.1087

[phy212880-bib-0025] Kawada, T. , T. Miyamoto , K. Uemura , K. Kashihara , A. Kamiya , M. Sugimachi , et al. 2004 Effects of neuronal norepinephrine uptake blockade on baroreflex neural and peripheral arc transfer characteristics. Am. J. Physiol. Regul. Integr. Comp. Physiol. 286:R1110–R1120. 14962824 10.1152/ajpregu.00527.2003

[phy212880-bib-0026] Kawada, T. , M. Li , A. Kamiya , S. Shimizu , K. Uemura , H. Yamamoto , et al. 2010 Open‐loop dynamic and static characteristics of the carotid sinus baroreflex in rats with chronic heart failure after myocardial infarction. J. Physiol. Sci. 60:283–298. 20514557 10.1007/s12576-010-0096-9 PMC10717991

[phy212880-bib-0027] Kawada, T. , T. Akiyama , S. Shimizu , Y. Sata , M. J. Turner , M. Shirai , et al. 2014a Acute effects of arterial baroreflex on sympathetic nerve activity and plasma norepinephrine concentration. Auton. Neurosci. 186:62–68. 25458434 10.1016/j.autneu.2014.10.016

[phy212880-bib-0028] Kawada, T. , M. Li , C. Zheng , S. Shimizu , K. Uemura , M. J. Turner , et al. 2014b Chronic vagal nerve stimulation improves baroreflex neural arc function in heart failure rats. J. Appl. Physiol. 116:1308–1314. 24674859 10.1152/japplphysiol.00140.2014

[phy212880-bib-0029] Kawada, T. , M. Li , Y. Sata , C. Zheng , M. J. Turner , S. Shimizu , et al. 2015 Calibration of baroreflex equilibrium diagram based on exogenous pressor agents in chronic heart failure rats. Clin. Med. Insights Cardiol. 9(Suppl. 1):1–9. 25698884 10.4137/CMC.S18759 PMC4319654

[phy212880-bib-0030] Kent, B. B. , J. W. Drane , B. Blumenstein , and J. W. Manning . 1972 A mathematical model to assess changes in the baroreceptor reflex. Cardiology 57:295–310. 4651782 10.1159/000169528

[phy212880-bib-0031] Kvetňanský, R. , K. Pacák , L. Sabban , I. J. Kopin , and D. S. Goldstein . 1998 Stressor specificity of peripheral catecholaminergic activation. Adv. Pharmacol. 42:556–560. 9327962 10.1016/s1054-3589(08)60811-x

[phy212880-bib-0032] Leenen, F. H. 2007 Brain mechanisms contributing to sympathetic hyperactivity and heart failure. Circ. Res. 101:221–223. 17673679 10.1161/CIRCRESAHA.107.158261

[phy212880-bib-0033] Li, M. , C. Zheng , T. Sato , T. Kawada , M. Sugimachi , and K. Sunagawa . 2004 Vagal nerve stimulation markedly improves long‐term survival after chronic heart failure in rats. Circulation 109:120–124. 14662714 10.1161/01.CIR.0000105721.71640.DA

[phy212880-bib-0034] Li, M. , C. Zheng , T. Kawada , M. Inagaki , K. Uemura , and M. Sugimachi . 2014 Adding the acetylcholinesterase inhibitor, donepezil, to losartan treatment markedly improves long‐term survival in rats with chronic heart failure. Eur. J. Heart Fail. 16:1056–1065. 25201490 10.1002/ejhf.164

[phy212880-bib-0035] Liu, J. L. , and I. H. Zucker . 1999 Regulation of sympathetic nerve activity in heart failure: a role for nitric oxide and angiotensin II. Circ. Res. 84:417–423. 10066676 10.1161/01.res.84.4.417

[phy212880-bib-0036] Lohmeier, T. E. , R. Iliescu , T. M. Dwyer , E. D. Irwin , A. W. Cates , and M. A. Rossing . 2010 Sustained suppression of sympathetic activity and arterial pressure during chronic activation of the carotid baroreflex. Am. J. Physiol. Heart Circ. Physiol. 299:H402–H409. 20511410 10.1152/ajpheart.00372.2010 PMC2930387

[phy212880-bib-0037] Ma, X. , C. D. Sigmund , S. D. Hingtgen , X. Tian , R. L. Davisson , F. M. Abboud , et al. 2004 Ganglionic action of angiotensin contributes to sympathetic activity in renin‐angiotensin transgenic mice. Hypertension 43:312–316. 14699002 10.1161/01.HYP.0000111835.16662.43

[phy212880-bib-0038] Majewski, H. , M. J. Rand , and L. H. Tung . 1981 Activation of prejunctional *β*‐adrenoceptors in rat atria by adrenaline applied exogenously or released as a co‐transmitter. Br. J. Pharmacol. 73:669–679. 6113865 10.1111/j.1476-5381.1981.tb16802.x PMC2071700

[phy212880-bib-0039] Nicholls, D. G. 1994 Proteins, Transmitters and Synapses. UK. Blackwell Sciences, Oxford.

[phy212880-bib-0040] Péronnet, F. , G. Boudreau , J. de Champlain , and R. Nadeau . 1994 Effect of changes in myocardial epinephrine stores on plasma norepinephrine gradient across the dog heart. Am. J. Physiol. 266:H2404–H2409. 7912901 10.1152/ajpheart.1994.266.6.H2404

[phy212880-bib-0041] Pfeffer, J. M. , M. A. Pfeffer , P. J. Fletcher , and E. Braunwald . 1991 Progressive ventricular remodeling in rats with myocardial infarction. Am. J. Physiol. 260:H1406–H1414. 2035662 10.1152/ajpheart.1991.260.5.H1406

[phy212880-bib-0042] Pitt, B. , P. A. Poole‐Wilson , R. Segal , F. A. Martinez , K. Dickstein , A. J. Camm , et al. 2000 Effect of losartan compared with captoril on mortality in patients with symptomatic heart failure: randomized trial–the Losartan Heart Failure Survival Study ELITE II. Lancet 355:1582–1587. 10821361 10.1016/s0140-6736(00)02213-3

[phy212880-bib-0043] Reid, I. A. 1992 Interactions between ANG II, sympathetic nervous system, and baroreceptor reflexes in regulation of blood pressure. Am. J. Physiol. 262:E763–E778. 1616014 10.1152/ajpendo.1992.262.6.E763

[phy212880-bib-0044] Sata, Y. , T. Kawada , S. Shimizu , A. Kamiya , T. Akiyama , and M. Sugimachi . 2015 Predominant role of neural arc in sympathetic baroreflex resetting of spontaneously hypertensive rats. Circ. J. 79:592–599. 25746544 10.1253/circj.CJ-14-1013

[phy212880-bib-0045] Sato, T. , T. Kawada , H. Miyano , T. Shishido , M. Inagaki , R. Yoshimura , et al. 1999 New simple methods for isolating baroreceptor regions of carotid sinus and aortic depressor nerves in rats. Am. J. Physiol. Heart Circ. Physiol. 276:H326–H332. 10.1152/ajpheart.1999.276.1.H326 9887047

[phy212880-bib-0046] Schömig, A. , S. Fischer , T. Kurz , G. Richardt , and E. Schömig . 1987 Nonexocytotic release of endogenous noradrenaline in the ischemic and anoxic rat heart: mechanism and metabolic requirements. Circ. Res. 60:194–205. 3568291 10.1161/01.res.60.2.194

[phy212880-bib-0047] Schwartz, J. H. 2000 Neurotransmitters Pp. 280–297 in Kandel E. R., Schwartz J. H. and Jessell T. M., eds. Principles of Neural Science. 4th ed. McGraw‐Hill, New York.

[phy212880-bib-0048] Shimizu, S. , T. Akiyama , T. Kawada , T. Shishido , M. Mizuno , A. Kamiya , et al. 2010 In vivo direct monitoring of interstitial norepinephrine levels at the sinoatrial node. Auton. Neurosci. 152:115–118. 19767249 10.1016/j.autneu.2009.08.017

[phy212880-bib-0049] Shoukas, A. A. , C. A. Callahan , J. M. Lash , and E. B. Haase . 1991 New technique to completely isolate carotid sinus baroreceptor regions in rats. Am. J. Physiol. Heart Circ. Physiol. 260:H300–H303. 10.1152/ajpheart.1991.260.1.H300 1992808

[phy212880-bib-0050] Svensson, T. H. , and T. Usdin . 1978 Feedback inhibition of brain noradrenaline neurons by tricyclic antidepressants: *α*‐receptor mediation. Science 202:1089–1091. 213833 10.1126/science.213833

[phy212880-bib-0051] Thrasher, T. N. 2002 Unloading arterial baroreceptors causes neurogenic hypertension. Am. J. Physiol. Regul. Integr. Comp. Physiol. 282:R1044–R1053. 11893608 10.1152/ajpregu.00431.2001

[phy212880-bib-0052] Turner, M. J. , T. Kawada , S. Shimizu , M. Fukumitsu , and M. Sugimachi . 2015a Open‐loop characteristics of the arterial baroreflex after blockade of unmyelinated baroreceptors with resiniferatoxin. Auton. Neurosci. 193:38–43. 26049262 10.1016/j.autneu.2015.05.008

[phy212880-bib-0053] Turner, M. J. , T. Kawada , S. Shimizu , M. Fukumitsu , and M. Sugimachi . 2015b Differences in the dynamic baroreflex characteristics of unmyelinated and myelinated central pathways are less evident in spontaneously hypertensive rats. Am. J. Physiol. Regul. Integr. Comp. Physiol. 309:R1397–R1405. 26377561 10.1152/ajpregu.00315.2015

[phy212880-bib-0054] Victor, R. G. 2015 Carotid baroreflex activation therapy for resistant hypertension. Nat. Rev. Cardiol. 12:451–463. 26149485 10.1038/nrcardio.2015.96

[phy212880-bib-0055] Viquerat, C. E. , P. Daly , K. Swdberg , C. Evers , D. Curran , W. W. Parmley , et al. 1985 Endogenous catecholamine levels in chronic heart failure. Relation to the severity of hemodynamic abnormalities. Am. J. Med. 78:455–460. 3976704 10.1016/0002-9343(85)90338-9

[phy212880-bib-0056] Wang, W. , J. S. Chen , and I. H. Zucker . 1990 Carotid sinus baroreceptor sensitivity in experimental heart failure. Circulation 81:1959–1966. 2344687 10.1161/01.cir.81.6.1959

[phy212880-bib-0057] White, C. W. 1981 Abnormalities in baroreflex control of heart rate in canine heart failure. Am. J. Physiol. 240:H793–H799. 7235038 10.1152/ajpheart.1981.240.5.H793

[phy212880-bib-0058] Yamaguchi, I. , and I. J. Kopin . 1980 Blood pressure, plasma catecholamines, and sympathetic outflow in pithed SHR and WKY rats. Am. J. Physiol. 238:H365–H372. 7369382 10.1152/ajpheart.1980.238.3.H365

[phy212880-bib-0059] Young, J. B. , R. M. Rosa , and L. Landsberg . 1984 Dissociation of sympathetic nervous system and adrenal medullary responses. Am. J. Physiol. 247:E35–E40. 6742188 10.1152/ajpendo.1984.247.1.E35

